# Nanosilicates in Compatibilized Mixed Recycled Polyolefins: Rheological Behavior and Film Production in a Circular Approach

**DOI:** 10.3390/nano11082128

**Published:** 2021-08-20

**Authors:** Emilia Garofalo, Luciano Di Maio, Paola Scarfato, Annalisa Apicella, Antonio Protopapa, Loredana Incarnato

**Affiliations:** 1 Department of Industrial Engineering, University of Salerno, Via Giovanni Paolo II, 84084 Fisciano, Italy; egarofalo@unisa.it (E.G.); pscarfato@unisa.it (P.S.); anapicella@unisa.it (A.A.); lincarnato@unisa.it (L.I.); 2 COREPLA-Italian Consortium for the Collection and Recycling of Plastic Packages, Via del Vecchio Politecnico, 20121 Milano, Italy; protopapa@ext.corepla.it

**Keywords:** post-consumer plastic films, mixed polyolefins, mechanical recycling, film blowing, extensional rheology

## Abstract

Currently, plastic packaging represents a global challenge and has become a key point of attention for governments, media and consumers due to the visibility of the waste it generates. Despite their high resource efficiency, the perceived non-recyclability of polymeric films risks precluding them from being a relevant packaging solution in a circular economy approach. In this regard, the aim of this study was to implement a strategy to try closing the loop, via the mechanical recycling of post-consumer flexible packaging of small size (denoted as Fil-s) to obtain new films. In particular, two lots of Fil-s were used, which are PE/PP blends differing for the PP content and the presence of polar contaminants. The suitability for film blowing extrusion of these recycled materials, as such and after the addition of a compatibilizer and/or a lamellar nanosilicate, was evaluated. It was first evidenced that the difficulty of producing blown films with the pristine recycled materials, due to the frequent bubble breakages, occurring even at low draw ratios. Moreover, the shear and extensional rheological behavior of all Fil-s based systems was usefully correlated with their processability features, evidencing the key roles of the nanofiller to stabilize the bubble and of the compatibilizer to ensure a uniform film deformation, avoiding its premature breakage. Even if the adopted upgrading strategies allowed the production of blown films with both types of Fil-s, the different components of the recycled matrices were proven to significantly affect their processability and final film performances.

## 1. Introduction

The circularity in the economy of plastic packaging today represents one of the biggest global challenges. To address this, the European Commission [1] has proposed ambitious recycling targets (up to 55% by 2030) for plastic packages. However, added value in a circular economy involves not only high recycling rates but parameters such as “resource efficiency” and “circularity” throughout the whole package lifecycle and the related environmental impact should also be taken into account [2].

In this regard, flexible films are highly material-efficient compared with other packaging formats, as is clearly evidenced by their measured “packaging to product ratio” [3]. Moreover, flexible packaging allows reducing space and weight in each transportation step along the supply chain, with consequent cost savings and reduction of the environmental impact, especially regarding the overall carbon footprint. Finally, by delivering an optimized fit-for-purpose packaging, plastic films contribute to shelf-life extension and food waste prevention. This is of particular relevance considering that for many foodstuffs, even with small pack sizes, the packaging accounts for less than 10% of the overall environmental impact across a range of LCA (life cycle assessment) indicators [2]. However, even if flexible packaging is proven to be very resource-efficient, it is not yet widely recycled, and this is perceived as a weakness in a circular economy model. To quantify the potential for recycling plastic films, first of all, it is important to have reliable information on the flexible packaging market volumes, as well as on the materials and structures mostly used.

Flexible packaging represents one-fifth (20%) of the Western European consumption of packaging (by value), and Europe uses one-third (33%) of the global plastic films production (by value) [4]. Moreover, whilst still needing more robust data, mono-material flexible packages (especially made of PE) seem dominant in the market, and this fraction, i.e., about 80% by weight [4], has excellent recycling potential if it was collected and sorted. This represents a necessary starting condition to increase the plastics recycling rates, in line with the European policy ambitions, and to create downstream market opportunities, as expected in a circular economy approach.

In this context, only in few European countries, among which there is Italy, the waste collection includes all plastic packaging in addition to those with greater added value, such as PET and HDPE rigid packaging. In particular, flexible packages from the urban waste collection are generally sorted into a separate stream, principally made of large PE films, or into the mixed plastics fraction, in the case of smaller films, including pouches and snack packaging. Moreover, it is worth pointing out that the sorting and recycling facilities in Italy are currently able to further subdivide the mixed polyolefin waste stream into higher-value products, such as PP films and small size films (Fil-s).

This latter fraction has been the objective of extensive experimental activity conducted by our research group in collaboration with COREPLA (the Italian Consortium for the Collection, Recycling and Recovery of Plastic Packaging). In particular, from our previous studies carried out on several lots of Fil-s, the mechanical recycling process of small-size films (lower than 0.125 m^2^) allows a secondary raw material (Fil-s) to be obtained, whose major fraction is constituted by polyethylene, with a lower percentage of PP (from 5 to 15% in weight) [5,6,7]. Other than the composition assessment of Fil-s, some compatibilization strategies, through a reactive extrusion and/or compounding with nanofillers, were also successfully implemented to improve the mechanical performances of this recycled material [5,6,7]. Moreover, the hygroscopicity issue of Fil-s was also addressed [8,9], and its final applications in the piping sector were evaluated [10].

In this research, the suitability of Fil-s for film blowing extrusion was investigated, using the recycled material as such and after proper upgrading steps. Regarding this latter aspect, the best compatibilization strategies, implemented with Fil-s in our previous works, were assessed individually and synergistically in order to attain high-quality recycled materials [11]. The aim was to recover as much as possible the value of post-consumer flexible packaging waste, from which Fil-s was obtained, following a circular approach, instead of a recycling cascade, where the secondary raw materials are used for alternative products with less demanding specifications. On the other hand, there is a great potentiality for the use of these recycled materials within the film market sectors [4], such as for secondary packaging to bind or wrap items together. Further applications concern agriculture (as silage films, mulch films and stretch films) and the building fields. Moreover, a recent study by Radusin et al. [12] deals with the use of recycled polyethylene (from post-consumer flexible packaging) as the mid-layer between two layers of virgin PE so that the resulting multilayer films are suitable for food contact applications.

Even if the several lots of Fil-s, analyzed in our previous studies [5,6,7,8,9,10], qualitatively showed a similar chemical composition, the detected differences in the polypropylene contents might affect the effectiveness of the compatibilization strategies and, in turn, Fil-s processability by film blowing extrusion, as well as the mechanical performances of the obtained films. Taking this into account, two Fil-s batches were considered in this research; specifically, the ones with the highest (ca 15%, denoted as Fil-s A) and the lowest (ca 5%, denoted as Fil-s B) measured PP percentages. The compounding of Fil-s with an experimental compatibilizer grafted with MA, alone and together with a lamellar nanosilicate, was first carried out by means of a twin-screw extruder. Successively, the effects of the compatibilizer and the nanofiller, both individually and simultaneously, on the processability of Fil-s through film blowing extrusion were investigated in the present study. In particular, the rheological behavior of the different Fil-s based systems was analyzed in shear and extensional modes, this latter being of fundamental relevance to foresee the suitability of polymeric materials for processes where stretching stages are involved. Finally, a laboratory-scale film blowing apparatus was used to extrude the several Fil-s based systems, and the obtained films were characterized through optical, thermal and tensile mechanical tests.

## 2. Materials and Methods

### 2.1. Materials

The recycled material used in this study and denoted as Fil-s was provided by COREPLA (Italian Consortium for the Collection and Recycling of Plastic Packages—Milan, Italy). It represents a secondary raw material, deriving from the mechanical recycling of post-consumer flexible films of small size (<0.125 m^2^), and it is principally made of mixed polyolefins, with very low amounts of polar contaminants. In particular, two different lots of Fil-s were analyzed: the polyolefin fraction of the first one (Fil-s A) is constituted by approximately 15 wt% of polypropylene, while the other one (Fil-s B) contains a minor fraction of PP, equal to about 5 wt%. Moreover, based on the thermogravimetric analysis (data not shown) of both the lots of Fil-s, an unburned residue at 600 °C of 3 ± 0.5 wt% was obtained.

The proprietary maleic anhydride (MA) grafted additive, made of a PE/PP blend with a PP amount lower than 5 wt%, was provided by the Italian company Auserpolimeri (Lucca, Italy). The percentage of maleic anhydride inside this additive (denoted in the following as PE/PP-g-MA) was equal to about 0.5 wt% [6,10].

A lamellar silicate, Dellite D67G (supplied by Laviosa Chimica Mineraria, Cagliari, Italy) that is organically modified by dimethyl dehydrogenated tallow (C14–C18) ammonium, was used as a nanofiller.

### 2.2. Compounding with the Compatibilizer and/or the Nanofiller and Film Blowing Extrusion

The PE/PP-g-MA compatibilizer and the nanofiller were melt compounded, individually and simultaneously, with both Fil-s batches using a twin-screw extruder (Dr. Collin GmbH—model ZK 25-48D, Munich, Germany) equipped with co-rotating intermeshing screws (D_screw_ = 25 mm, L/D = 42). Before the extrusion, all the materials were dried at 70 °C for 18 h in an oven under vacuum conditions. Fil-s pellets were first mechanically mixed with 5 wt% of the compatibilizer and/or 5 wt% of the lamellar silicate, and each blend was then fed inside the extruder by means of a volumetric feeder. A screw speed of 100 rpm was imposed, and a flat temperature profile of 190 °C was set along the barrel, while at the die, the temperature of 160 °C was used. The extruded strand was water-cooled and then pelletized.

The technical specifications of the laboratory-scale plant used for film blowing extrusion of Fil-s based systems are reported in Table 1.

A screw speed of 75 rpm was set during films extrusion for all the recycled systems analyzed, but different temperature profiles and stretching conditions (take-up ratio, TUR, and blow-up ratio, BUR) were imposed according to the selected Fil-s batches, as reported in Table 2.

### 2.3. Characterization Techniques

The thermal analysis of all Fil-s based systems was carried out with a DSC30 Mettler calorimeter (Mettler-Toledo International Inc., USA). A first heating scan at 10 °C/min from 0 to 250 °C was set, followed by an isotherm at 250 °C for 5 min (to remove the thermo-mechanical history of the samples), a cooling scan from 250 to 0 °C and a second heating scan from 0 to 250 °C at the same scan rate. For the determination of the crystallinity degrees of the samples, the values of ΔH_m_^0^ = 293 [13] and ΔH_m_^0^ = 207 J/g [14] were used as the melting enthalpies for 100% crystalline PE and for 100% crystalline PP, respectively. DSC tests were conducted in triplicate, and the standard deviations of the results were less than 5%.

A Nexus ThermoNicolet spectrometer (Thermo Scientific, Waltham, MA, USA) equipped with a SmartPerformer accessory for ATR measurements was used to perform Fourier transform infrared (FT-IR) spectroscopic analysis. The samples were scanned 64 times over the wavenumbers range from 4000 to 650 cm^−1^ and with a resolution of 2 cm^−1^.

Dynamic shear rheological experiments were carried out with a rotational rheometer ARES (Rheometric Scientific, New Castle, DE, USA), equipped with 25 mm diameter parallel plates. The tests were performed at 190 °C, under a nitrogen atmosphere and in an angular frequency range from 0.1 to 100 rad/s. A strain amplitude of 1% was used since it was proven to guarantee the linear viscoelastic regime during the shear rheological measurements.

Transient extensional rheological tests were performed with a Sentmanat Extensional Rheometer (model SER-HV-A01, manufactured by Xpansion Instruments, Tallmadge, OH, USA) [15,16], which was mounted on the ARES rotational rheometer. Samples with thickness, width and length of 0.8 × 6 × 15 mm, respectively, were prepared by means of a preheated hydraulic press. The measurements were carried out at 190 °C and at Hencky strain rates of 0.5, 1 and 10 s^−1^ until the maximum achievable Hencky strain (ca. 3.5). The rheological tests with the rotational rheometer (both in shear and in extensional mode) were conducted in triplicate, and standard deviations of less than 3% were obtained.

The steady shear rheological experiments were carried out with a Rosand RH7 capillary rheometer (Bohlin Instruments, Worcestershire, UK), equipped with a 1 mm diameter capillary die (L/D = 30). The measurements were performed at 190 °C, and each sample was tested in triplicate, with standard deviations below 5%.

A Zeiss EVO MA10 microscope (Carl Zeiss SMT AG, München-Hallbergmoos, Germany) was used to perform the scanning electron microscopy (SEM) analysis. Prior to capturing the images, the specimens were cryo-fractured in liquid nitrogen and sputter-coated with a 200–440 Å thick gold layer by means of a Leica EMSCD005 metallization device. 

The external surfaces of Fil-s based films were investigated using a Zeiss Axioskop microscope (Carl Zeiss Vision, Oberkochen, Germany).

Tensile mechanical tests were conducted with a CMT4000 Series dynamometer (SANS, Shenzhen, China), equipped with a load cell of 100 N, and according to ASTM D882 standard. Rectangular specimens were cut from the blown films along the machine direction and were tested at crosshead speeds of 5 and 500 mm/min to determine Young’s modulus and the properties (strain and stress) at break, respectively.

The tensile mechanical data were statistically analyzed by means of the one-way ANOVA technique, using the Data Analysis ToolPak of Microsoft Excel 2016. The significance level was always set to 0.05. Moreover, since the data groups were not numerous, the statistical analysis was implemented on pairs of samples, considering all the possible comparisons aimed to evaluate the effect of one variable at a time.

## 3. Results and Discussion

### 3.1. Main Differences between the Lots of Fil-s Selected for this Study

**Figure 1:**
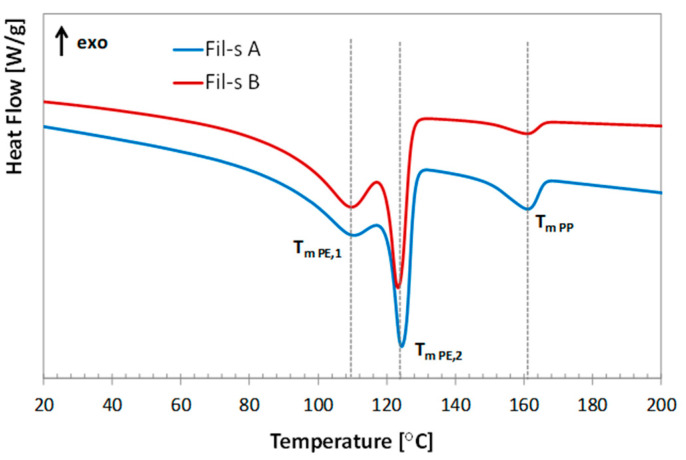
Second heating thermograms for the lots of Fil-s analyzed.

**Figure 2:**
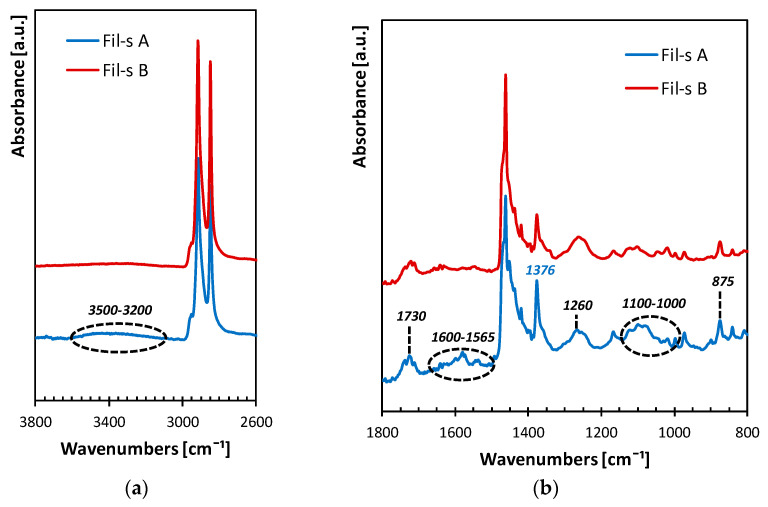
ATR-FTIR spectra for the lots of Fil-s analyzed in the wavenumbers intervals 3800–2600 (**a**) and 1800–800 (**b**).

Based on the previous research activity conducted on several batches of Fil-s and whose main results are reported in our recently published works [5,6,7,8,9,10], some differences in composition emerged among the lots of the recycled material analyzed, as expected considering the current difficulties in obtaining a clean and homogeneous waste stream from post-consumer flexible packaging of small size. In particular, in this study, we were concerned with two batches of Fil-s, denoted as Fil-s A and Fil-s B, whose second heating thermograms and FT-IR spectra are reported in Figure 1 and Figure 2, respectively, to highlight the principal differences between them, while we refer to our previous papers [5,6] for the extensive description of both the thermal and spectroscopic characteristics of Fil-s.

DSC plots for Fil-s A and Fil-s B show a first multiple peak (T_m PE,1_ ≅ 110 °C and T_m PE,2_ ≅ 124 °C), representative of the PE fraction, and a less pronounced one at a higher temperature (T_m PP_ ≅ 161 °C), corresponding to the PP component. In particular, the relative PE/PP amounts inside the several lots of Fil-s analyzed were determined using a calibration curve obtained from the thermal characterization of PE/PP model blends at different percentages of the virgin polymers [6]. In the case of the recycled materials used in this study, the main differences that can be deduced from the thermograms are: (i) the more intense PP peak for Fil-s A, which can be correlated to a polypropylene content of about 15 wt%, compared with Fil-s B, whose PP percentage (ca. 5 wt%) was significantly lower; (ii) a more pronounced shoulder of the PE peak (T_m PE,1_) for Fil-s B respect to Fil-s A, evidencing a major fraction of polyethylene with higher branch density (short and/or long branching) [17].

The FTIR-ATR plots in Figure 2 confirm that Fil-s A contains a higher amount of PP with respect to Fil-s B, as it can be clearly deduced from the more pronounced peak at 1376 cm^−1^, assigned to the scissoring of the methyl group CH_3_. The two lots of Fil-s also differ in the intensity of the other infrared bands highlighted in Figure 2, which can be related to the presence of polar contaminants, such as polyamide and polyester traces [6]. The assignments for these peaks are summarized in Table 3.

Many of the same absorption bands were also detected by Gala et al. [18], who conducted a detailed characterization of post-consumer plastic films, coming from mixed municipal solid waste, and correlated these signals to the presence of polymer additives (such as antioxidants, photo-stabilizers, flame retardants and so on) and/or to the photo-thermal oxidative phenomena occurring during the processing and the end-use stages of the polymeric materials. Regarding the recycled materials used in the present study, Fil-s A shows a higher number of polar contaminants compared with Fil-s B, in turn resulting in a more pronounced hygroscopic behavior. However, for both types of Fil-s it, was necessary to dehumidify the material before any extrusion operation was conducted during this research in order to be able to process these materials and to obtain products without macroscopic defects.

### 3.2. Compounding of Fil-s with the Nanofiller and/or the Compatibilizer

**Figure 3:**
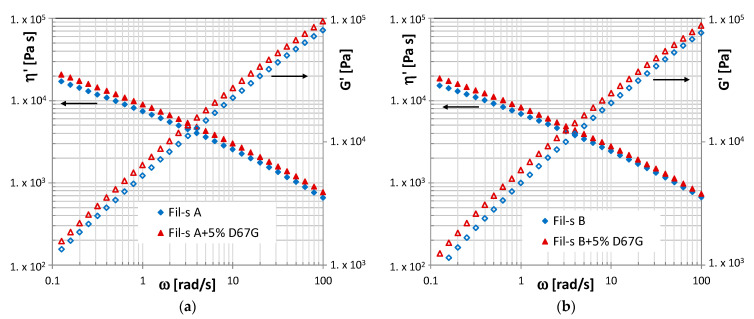
Comparison of the dynamic viscosity (η’) and storage modulus (G’) plots of Fil-s A (**a**) and Fil-s B (**b**) and the corresponding nanocomposite systems with Dellite 67G.

Both the lots of Fil-s were melt-compounded with 5 wt% of a layered nanofiller, and the resulting linear viscoelastic behavior, measured using dynamic shear rheological tests, is presented in Figure 3.

We already reported on the dynamic shear rheological behavior of Fil-s and its composites with an organically modified silicate [6], evidencing only a slight increase of the viscoelastic properties for the nanocomposites compared with the neat matrix due to a poor exfoliation degree of the filler inside the recycled material. This outcome was also confirmed in this study, with no significant differences observed for both the lots of Fil-s analyzed, despite the composition differences highlighted in the previous paragraph. In particular, the same trends of the dynamic viscosity and the viscoelastic modulus, as a function of the angular frequency, can be observed for both the recycled materials, with only a slight difference in the absolute values of these properties that resulted in higher Fil-s A, due to its bigger percentage of PP with respect to Fil-s B.

Conversely, the compositional peculiarities of the recycled materials used and, mainly the more pronounced polar character of Fil-s A, affected the rheological response in a different way after the addition of an experimental compatibilizer grafted with maleic anhydride (MA). This additive, obtained from the reactive extrusion with MA of a virgin PE/PP blend and denoted in the following as PE/PP-g-MA, was already successfully tested in our previous research to make Fil-s suitable for applications in the piping sector [10]. In the present study, its ability to improve not only the compatibility between the main polyolefin fractions of Fil-s but also the nanofiller dispersion inside the recycled materials has been assessed. Regarding the first function, the additive acts as a block and/or graft PE/PP copolymer inside Fil-s. In fact, as extensively reported in the literature for virgin polyethylene/polypropylene blends [19,20] and, as also successfully implemented by means of the reactive extrusion of Fil-s [6], the maleic anhydride is able to stabilize the macro-radicals’ sites formed during the reaction of the polymer with a peroxide. In other words, it creates an inter-chain block or graft copolymers, leading to enhanced compatibility and improved mechanical properties [21] of the obtained material. 

The alternative strategy for Fil-s upgrading, which foresees the addition of a PE/PP-g-MA compatibilizer, instead of the direct, reactive grafting/compatibilization with maleic anhydride of the recycled material, offers the twofold advantage of providing the plastics converters with a ready-to-use compatibilizer and of more easily tuning the compatibilization process. Concerning this latter issue, while it is well known that the inherent complexity of conducting free radical reactions for grafting MA on virgin polyolefin matrices, without extensive side reactions [20,21], many further difficulties arise when the material comes from the post-consumer polymeric waste stream, due to the unpredictable presence of several types of contaminants [6], which might affect the yield and effectiveness of the reaction.

**Figure 4:**
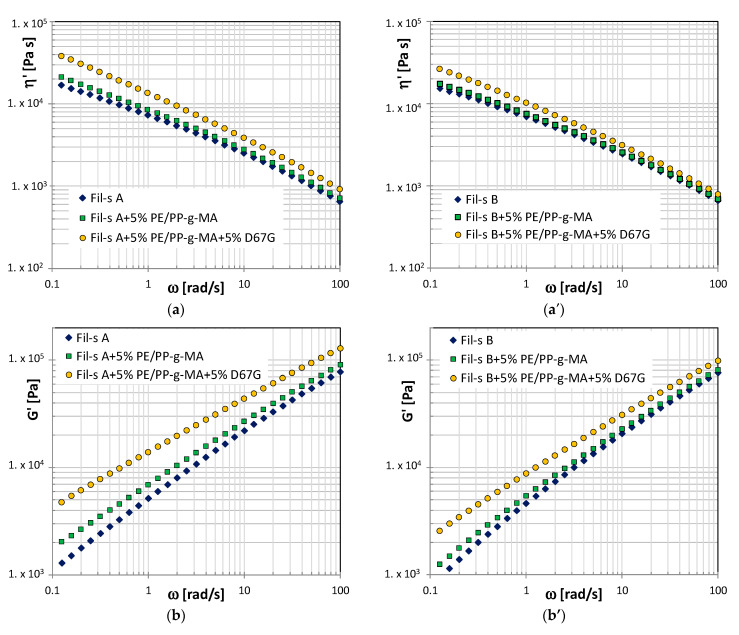

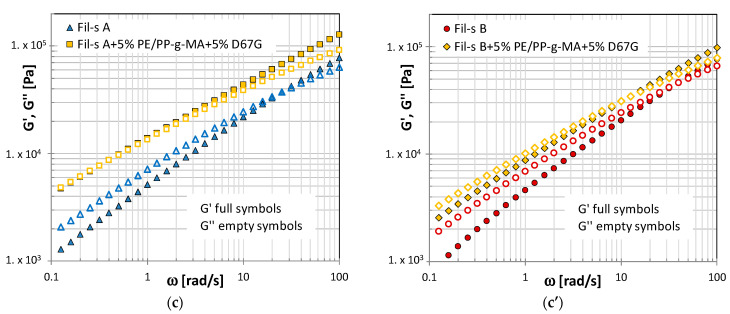
Comparison of the dynamic viscosity η’ (**a**,**a’**), the storage modulus plots G’ (**b**,**b**’) and the storage and loss moduli, G’, G”, plots (**c**,**c**’) for Fil-s A and Fil-s B-based systems, respectively.

The effect of the PE/PP-g-MA addition alone and simultaneously with the nanofiller on the linear viscoelastic behavior in the shear mode of both the Fil-s batches is shown in Figure 4.

**Figure 5:**
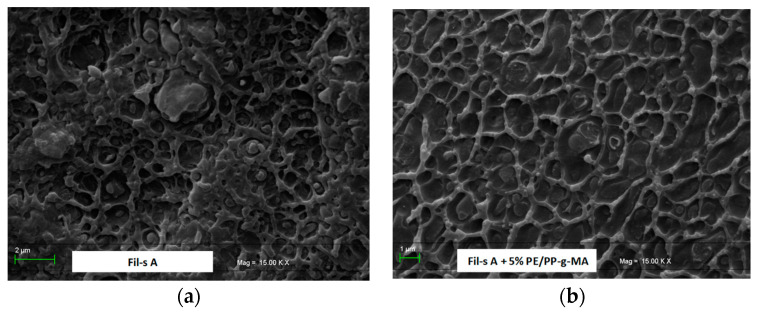
SEM images of (**a**) Fil-s A as such and (**b**) after the addition of PE/PP-g-MA compatibilizer.

In the low-frequency (ω) range, a slight increment of the dynamic viscosity, η’, and the storage modulus, G’, can be observed in both the systems due to the presence of the compatibilizer. This change in the low-frequency dynamics is more pronounced in the case of Fil-s A, and it can be attributed to a higher amount of contaminants, whose polar groups (–NH, C–O and –OH) establish hydrogen bonds with the MA molecules grafted on the compatibilizer chains. This results in the formation of a weak network that affects the viscoelastic behavior of the compatibilized recycled materials at low ω, and it is destroyed at higher frequencies [22]. The compatibilization effectiveness of PE/PP-g-MA additive is also proven by SEM micrographs (Figure 5a,b), captured on the sections of Fil-s A samples, before and after the compounding with the maleated additive. In fact, in the presence of the compatibilizer, the PP droplet inclusions look better embedded within the major PE fraction of the recycled material (Figure 5b) in comparison with Fil-s A without compatibilizer, where clear discontinuities can be observed at PE/PP interfaces (Figure 5a), as a consequence of poor compatibility between these polymeric fractions.

**Figure 6:**
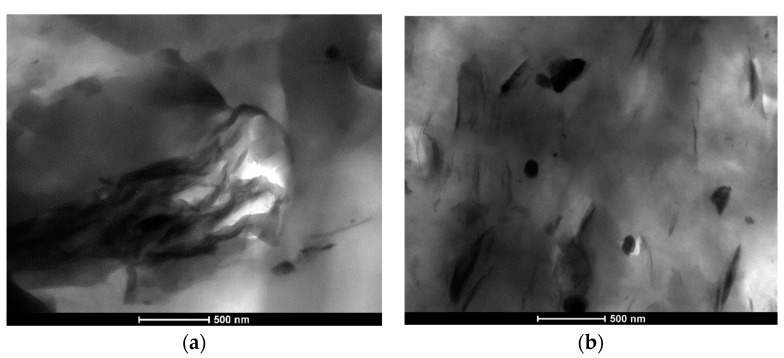
TEM images of (**a**) Fil-s A + 5% D67G and (**b**) Fil-s A + 5% PE/PP-g-MA +5% D67G.

Moreover, the presence of the PE/PP-g-MA additive strongly influences the viscoelastic behavior of both Fil-s systems filled with the nanosilicate (Figure 4), with significant increments of the dynamic viscosity η’ and the storage modulus G’ values compared with the pristine recycled materials. In particular, higher values of these viscoelastic properties and stronger incremental increases with respect to the neat matrix can be observed for the Fil-A-based system, suggesting a better polymer-silicate interaction. This latter outcome can be correlated to the finer distribution of the PE/PP-g-MA additive inside Fil-s A with respect to Fil-s B due to its higher polar character, as demonstrated in the previous paragraph. In turn, this affects the filler dispersion degree, as also evidenced by TEM micrographs of Fil-s A-based composites in Figure 6a,b.

In fact, the presence of the PE/PP-g-MA additive allows a better dispersion of the nanofiller to be obtained, with smaller stacks of the layered silicate and some exfoliated platelets, compared with the system without the compatibilizer, which shows filler aggregates of micrometric size. These results are in line with the large scientific literature [23,24,25] on virgin polymer-layered silicate nanocomposites that have highlighted the strict correlation existing between the dynamic shear rheological behavior of these composites and their nanostructure.

Moreover, the typical transition from a liquid-like behavior of the pristine polymer matrices to a solid-like one for the corresponding nanocomposites, as a consequence of the strong interactions among nanoparticles and/or lamellar silicate-polymer chains, can also be observed in Figure 4c,c’, looking at the trends of the storage G’ and loss moduli G” for both the Fil-s hybrid systems. The structural network formed in well-dispersed nanocomposites strongly affects the storage modulus (G’) since it is very responsive (in the low-frequency range) to the material morphological state, evidencing a reduced dependence on ω as the nanoplatelets dispersion degree increases. 

In this regard, it can be observed that the sample Fil-s A + 5% PE/PP-g-MA + 5% D67G shows the highest storage modulus values and the corresponding G’ and G” curves overlap within the two low-frequency decades investigated, with G’ becoming higher at ω > 10 rad/s. Moreover, in order to quantitatively compare the effect of the formed nanostructure on the storage modulus plots for all the samples analyzed, the G’ slopes at low angular frequencies (known as the terminal regime) are reported in Table 4.

These data highlight that, even for the pristine recycled materials, the G’ slope deviates from the power-law dependence of two expected for a monodisperse entangled linear polymer melt in the terminal region, and it progressively becomes lower by adding the nanofiller, the compatibilizer and both of them. While the values of G’ slope for the neat Fil-s, lots can be related to a quite large polydispersity index, which is expected in the case of recycled materials [26,27], the further reduced slope, particularly for the compatibilized nanocomposite systems based on Fil-s A, can be attributed to the formation of supramolecular structures, due to non-covalent silicate–silicate, polymer–polymer and/or silicate–polymer interactions.

### 3.3. Processability by Film Blowing of Fil-s Based Systems

The processability of a polymer by the film blowing process should be considered in two respects. First of all, low pressures in the extruder and the lack of melt instabilities [28] are desirable in the extrusion step, while good bubble stability and film homogeneity, even at high draw-down ratios, represent necessary conditions in order to obtain a satisfactory film quality and thus good final performances.

**Figure 7:**
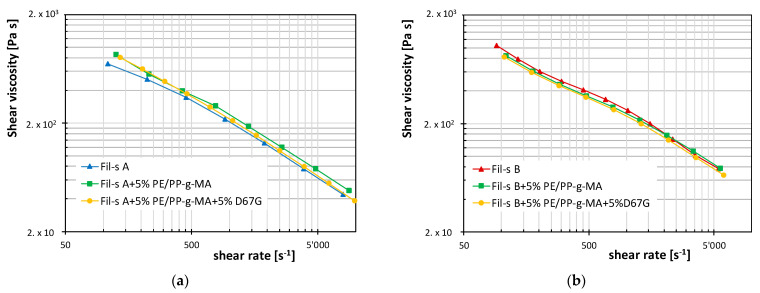
Shear viscosity vs. shear rate plots for (**a**) Fil-s A-based systems and (**b**) Fil-s B-based systems. The solid lines do not represent model fittings, but they are reported to help data visualization.

Regarding the extrusion step, the shear-thinning behavior of the resins, at rates occurring during the extrusion process (around 100 s^−1^), is the most important requirement. Thus, the shear viscosity plots for both Fil-s lots and the corresponding systems with the compatibilizer and the nanofiller are reported as a function of shear rates in Figure 7.

Coherently with the trends of the dynamic rheological data, a pronounced shear-thinning behavior can be observed at high shear rates for all the systems analyzed, with only slight differences in the viscosity (η) values between the pristine recycled materials and the compatibilized and nanocomposite samples. In particular, in the case of Fil-s A, the presence of the PE/PP-g-MA additive determines a light increase of η, while an opposite behavior can be observed for the corresponding system based on Fil-s B. The further addition of the nanofiller inside both the compatibilized recycled materials has the effect of emphasizing the shear-thinning behavior of the systems, probably due to the nanoplatelets’ orientation in the flow direction [29].

It is now well established in the literature that, during the film-forming step, the bubble stability and melt extensibility are strongly affected by the elongational rheological behavior of the polymer [30,31,32,33,34,35,36]. 

In this study, transient extensional viscosity measurements were performed at Hencky strain rates of 0.5, 1 and 10 s^−1^ and attaining a maximum Hencky strain of about 3.5. This latter parameter comes close to that corresponding to a take-up ratio of 40 [34], and the selected elongational rates represent reasonable values within the rate distribution occurring during the film blowing process. In fact, the bubble is characterized by a wide distribution of Hencky strain rates [37], but they are within the range of the extensional rheological measurements carried out in this work 

**Figure 8:**
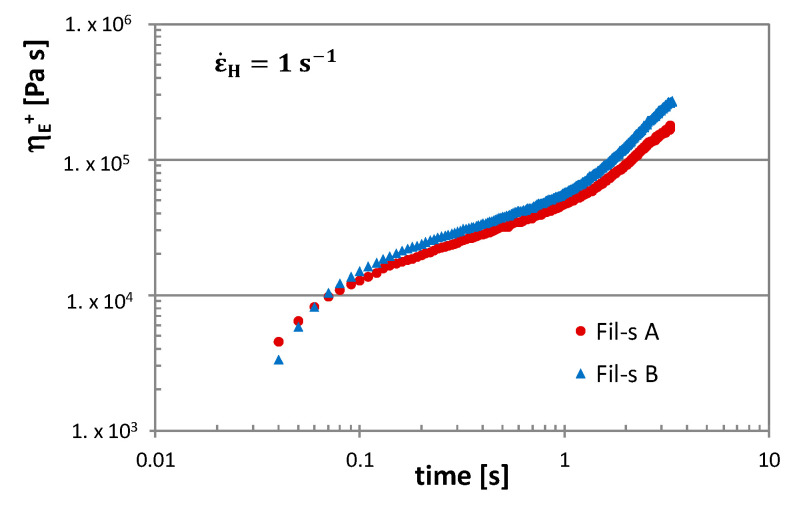
Tensile stress growth coefficient η_E_^+^ plots as a function of time at 190 °C and ${\dot{\varepsilon }}_{H}$ = 1 s^−1^ for Fil-s A and Fil-s B.

In Figure 8, the tensile stress growth coefficient (η_E_^+^) curves, as a function of time, are reported at 190 °C and at Henchy strain rate of 1 s^−1^ for both types of Fil-s. These plots show the typical trend observed for branched polyolefin resins with a linear extensional region at lower times, followed by a second zone, where a rapid upward of η_E_^+^ can be observed. This latter phenomenon is known as strain hardening, and it occurs in the molten recycled materials, mainly due to the presence of the polyethylene fraction with long-chain branches, which produce additional stress for the stretching of the macromolecules in the flow direction [30,38]. In particular, Fil-s B shows a more pronounced strain hardening behavior compared with Fil-s A, which is coherent with their compositional differences evidenced by DSC and FTIR analysis (Figure 1 and Figure 2). In fact, Fil-s B presents a major fraction of polyethylene with a higher branching density and a lower content of PP. This latter shows a poor strain hardening behavior since polypropylene grades traditionally used in the packaging sector possess a linear molecular structure and are intended for cast film extrusion.

Since the transient elongational rheological data, as a consequence of the addition of the compatibilizer and/or the nanofiller inside the recycled materials, are slightly different in their absolute values for the two batches of Fil-s analyzed but essentially follow the same trends, the stress growth coefficient curves are reported only for Fil-s B to avoid redundant data.

**Figure 9:**
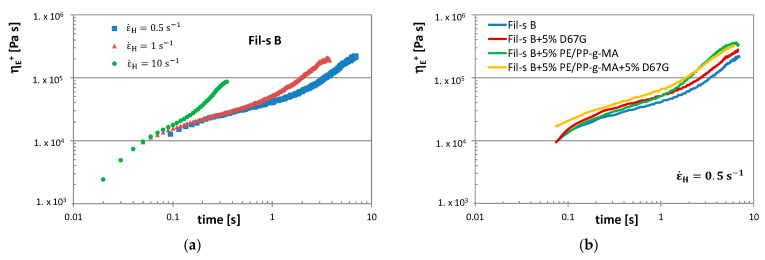

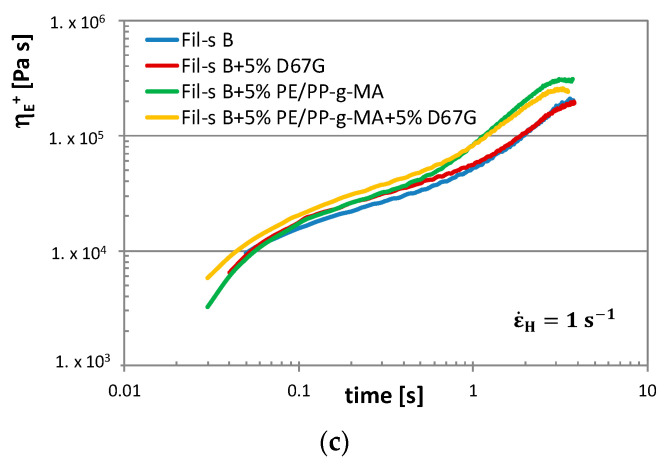
The tensile stress growth coefficients, η_E_^+^, as a function of time and at different Henchy strain rates (0.5, 1 and 10 s^−1^) are compared for Fil-s B (**a**) and for Fil-s B, Fil-s B + 5% PE/PP-g-MA, Fil-s B + 5% D67G and Fil-s B + 5% PE/PP-g-MA + 5% D67G at Henchy strain of 0.5 s^−1^ (**b**) and at Henchy strain of 1 s^−1^ (**c**).

In Figure 9, the η_E_^+^ plots of Fil-s B, at 190 °C and at different Henchy strain rates, are compared with the corresponding graphs of the compatibilized and nanocomposite Fil-s B-based systems.

First of all, it can be observed (Figure 9a) that the strain hardening behavior, exhibited by Fil-s B, is more pronounced by increasing the Henchy strain rates, as also happens in the case of virgin LDPE [39]. Moreover, by comparing the η_E_^+^ plots of all Fil-s B-based systems at a constant Henchy strain rate (Figure 9b,c), some interesting insights about their extensional behavior can be deduced.

The addition of the nanofiller in the recycled material determines an increment of the stress growth coefficient in the time range investigated, even if this effect is significantly reduced at the higher Henchy strain rates as a consequence of the silicate platelets orientation along the stretching direction [40,41]. Conversely, the presence of the PE/PP-g-MA compatibilizer inside Fil-s B causes a more pronounced strain hardening behavior compared with the pristine material, and this outcome appears almost unaltered by increasing strain rates. 

The simultaneous addition of the compatibilizer and the nanofiller turns out in the translation of the η_E_^+^ curve towards higher values of the stress growth coefficient, in comparison with both the neat Fil-s B and the uncompatibilized composite system Fil-s B + 5% D67G. This outcome can be attributed to the better nanoclay dispersion inside the recycled material due to the presence of the MA-grafted additive, as already demonstrated by the dynamic shear rheological data of the same systems (Figure 4). Moreover, the observed increments of η_E_^+^ are only slightly reduced at higher Henchy strain rates.

Many authors established useful correlations between the rheological behavior of virgin polyolefins (and their blends) and their processability characteristics during film blowing extrusion [30,31,32,33,34,35,36]. In this regard, it has been demonstrated that the potentiality of a polymer melt to be stretched into very thin films without breaking can be related to lower melt elasticity, measured in shear, and a smaller strain hardening in uniaxial elongational flow [30,42]. Conversely, good bubble stability is associated with the extension thickening (strain hardening) behavior of the material. 

To quantitatively correlate the strain hardening degree of a resin with its processability by film blowing, Jones and Kurtz [43] have defined a parameter, denoted as Extensional Viscosity Index (EVI), which is expressed by the following Equation (1).

(1) $$\mathrm{EVI}=\frac{\eta_{E}^{+}\left(\varepsilon_{H}=2.0;\;{\dot{\varepsilon }}_{H}=1{\;s}^{-1}\right)}{\eta_{E}^{+}\left(\varepsilon_{H}=0.2;{\dot{\varepsilon }}_{H}=1{\;s}^{-1}\right)}$$

In particular, a low-strain hardening material can be referred to as one for which the EVI value is less than 4.5. Several commercial LLDPEs with EVI’s less than 3.5 fall in this category and are able to achieve high drawability. However, the EVI parameter can be as high as 7 or 8 for commercial LDPEs that show better bubble stability with respect to HDPE and LLDPE resins.

To compare the processing behavior by film blowing of the recycled materials with the one of the virgin polyolefins, the EVI values were also calculated for all Fil-s B-based systems (Table 5).

From the data reported in Table 3, it can be deduced that for the specimens with the compatibilizer (Fil-s B + 5%PE/PP-g-MA and Fil-s B + 5%PE/PP-g-MA + 5%D67G) better bubble stability should be expected. However, another fundamental aspect has to be taken into account during film extrusion, which concerns the thickness uniformity of the blown films being important for their end-use properties. In this regard, in the absence of strain hardening, the thinner zone of a film will be further stretched due to the concentration of higher stress, and it will finally break. Conversely, for a material that shows strain hardening behavior, the rapid upward of η_E_^+^ with strain, resulting in a greater resistance to flow, will counteract any localized thinning of the sample and will assure its more uniform deformation [30].

### 3.4. Production of Blown Films with Fil-s Based Systems

Both Fil-s A and Fil-s B-based systems were extruded by means of a laboratory-scale film blowing apparatus in order to practically assess the suitability of these recycled materials (as such and after the compounding with the compatibilizer and/or the nanofiller) for flexible film production, with the aim to close the loop according to a circular economy approach.

During the process, it was necessary to set different operative conditions, in terms of temperature profile and stretching parameters, for the systems based on the two types of Fil-s, as reported in detail in the experimental section of the manuscript. In particular, in the case of Fil-s A, higher extrusion temperatures were set since it contains a major content of PP and of high-melting polar contaminants (such as polyesters and polyamides) compared with Fil-s B. The higher compositional dis-uniformity of Fil-s A also prevented the same degree of stretching set for Fil-s B-based systems to be achieved; therefore, resulting in thicker films (100 µm for Fil-s A samples against ca. 50 µm for Fil-s B specimens).

The main critical issue during film blowing extrusion concerns the onset of bubble instabilities since these phenomena do not allow uniform films with good final performances to be obtained, and, furthermore, they limit the process productivity. In this study, the bubble stability was visually verified, mainly looking at the duration of stability at given processing parameters (BUR and TUR). In this regard, the difficulty of producing blown films with both the pristine recycled materials was detected, due to frequent breakages occurring, even at low draw ratios. The processability of both the recycled materials significantly improved with the addition of the nanofiller, which allowed a very stable bubble to be obtained. Thisis coherent with the higher elasticity (as deduced by the storage modulus data in shear, Figure 4b,b’) and the larger stress growth coefficient values (Figure 9b) of the composites with respect to the neat matrices. 

On the other hand, the presence of PE/PP-g-MA additive significantly increased the strain-hardening character of the recycled materials (Figure 9), positively affecting the film deformation mechanism, so that in the case of Fil-s B + 5%PE/PP-g-MA system, it was possible to produce films with a very low thickness (about 30 µm). As clearly explained by Münstedt [30], the self-healing effect allows for a more homogeneous deformation of the samples that show a pronounced strain-hardening behavior. This effect is particularly beneficial during the stretching of recycled polymers since the presence of several impurities may cause the premature breakage of the samples. In fact, due to stress concentration at these spots, areas with a smaller section than the surrounding material are formed in their proximity, thus suffering a higher strain. If the specimen shows a strain hardening behavior, it is able to offer a higher resistance against further deformation, preventing the film breakage. 

The simultaneous addition of the compatibilizer and the nanofiller inside the recycled matrices ensured very good bubble stability but conversely reduced the material’s drawability with respect to the systems with the compatibilizer alone due to the higher shear and elongational viscosity values of the well-dispersed nanocomposite system.

The collected films were then characterized through optical, thermal and tensile mechanical tests.

**Figure 10:**
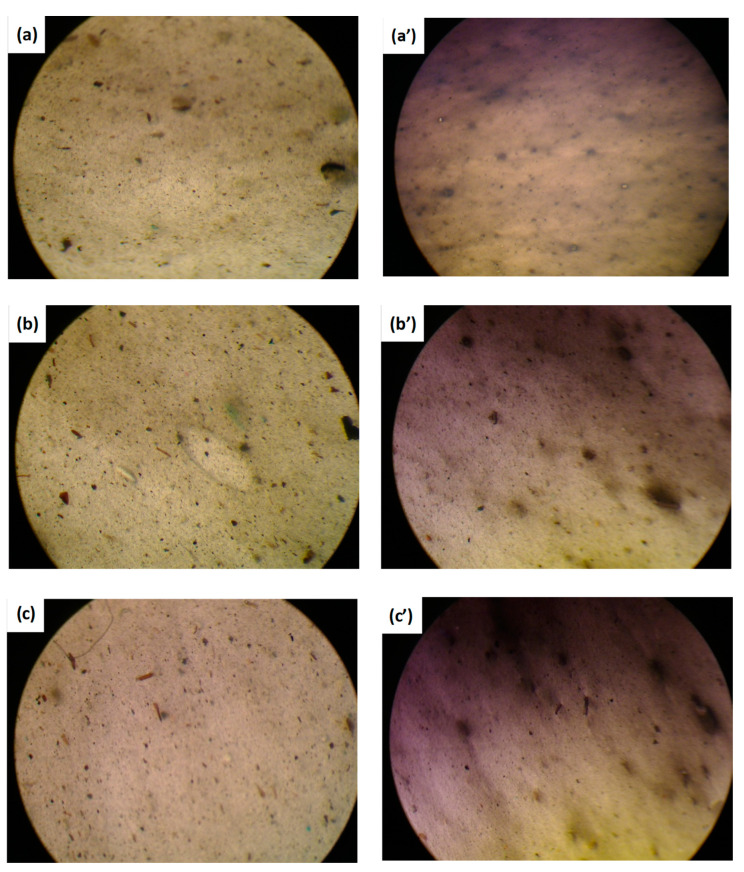
Images of the produced films (on the left Fil-s A-based systems and on the right Fil-s B-based systems) captured with an optical microscope at 8× magnification: (**a**,**a’**) Fil-s + 5%PE/PP-g-MA; (**b**,**b’**) Fil-s + 5%D67G; (**c**,**c’**) Fil-s + 5%PE/PP-g-MA + 5%D67G.

The surface of the different blown films was observed with an optical microscope in transmission mode, and the images, captured with 8x magnification, are compared in Figure 10. Some microscopic inclusions are clearly visible in all the samples, and next to these black spots, lighter zones can be observed, indicating thinner regions of the films due to the major deformations suffered.

The produced films were also characterized by means of DSC measurements, and the principal thermal parameters, relative to the first heating scan, for both Fil-s A and Fil-s B-based systems are reported in Table 6. No significant changes were evidenced in the main melting temperatures (T_mPE__,__2_ and T_mPP_) for both types of recycled materials following the addition of the nanofiller and/or the compatibilizer, but some considerations about films’ crystallinity degree should be pointed out, in order to coherently correlate the mechanical performances of the produced samples with the values of this parameter. In particular, it is important to take into account X_cPE_ data since the polyethylene fraction is the most abundant in both recycled materials.

First of all, some differences can be detected between the crystallinity degrees for Fil-s A-based systems and Fil-s B ones, with the latter showing lower X_c,PE_ values on average. Even if, during the film extrusion of both types of recycled materials, the same operative cooling parameters were set, the lower crystallinity of Fil-s B-based films can be related to their smaller thickness that allowed a faster cooling. Looking at the specimens with the same thickness, Fil-s B-based films show similar X_c,PE_ data, while in the case of Fil-s A-based films, only for the sample Fil-s A + 5%PE/PP-g-MA + 5%D67G a lower crystallinity degree can be observed. This outcome can be explained considering the effect of the nanoscale silicate dispersion on the macromolecules’ dynamics (Figure 4c), which results in slowing down the growth phase of the crystalline nuclei [44].

The final performances of the recycled films were evaluated in terms of tensile mechanical properties measured in the machine direction. These results are summarized in Table 7, grouping the films into sections. The data of the samples in each section were analyzed using the one-way ANOVA technique in order to evaluate whether the mean differences between these specimens are statistically significant.

Fil-s A-based films show higher values of Young’s modulus and strain at break compared with the corresponding specimens based on Fil-s B. While the greater stiffness data can be attributed to the presence of the major PP content inside Fil-s A, the higher values of deformation at break can be mainly related to the films’ thickness, which is about twice Fil-s B ones for Fil-s A systems.

Concerning Fil-s A-based films, the specimens with the silicate or the PE/PP-g-MA additive alone show similar (*p* > 0.05) Young’s modulus and strain at break properties, while the simultaneous addition of the compatibilizer and the nanofiller determines a significant stiffness reduction (about 15%) and a ductility increase (about 35% with respect to the film Fil-s A + 5%PE/PP-g-MA), which is coherent with the lower crystallinity degree of this sample.

In the case of Fil-s B-based systems, all the collected films exhibit similar strain at break properties (*p* > 0.05), while the nanocomposite samples show higher stiffness (about 20–30%) compared with the unfilled one (Fil-s B + 5%PE/PP-g-MA). However, regarding this latter film, a significant increment (*p* < 0.05) of both Young’s modulus (ca. 45%) and the tensile strength (ca. 80%) can be observed by increasing the stretching ratio in the machine direction. The improved mechanical performances of this thinner film, with respect to the thicker one, can be reasonably attributed to the resulting higher degree of macromolecules’ orientation in the stretching direction.

## 4. Conclusions

In this study, the suitability of a complex plastic packaging waste stream, constituted by post-consumer flexible films of small size (Fil-s), for film blowing extrusion was assessed. In particular, since the current sorting and recycling facilities do not allow lots of Fil-s with constant and repeatable features to be obtained, two batches of this recycled material were selected; specifically, the ones with the highest (ca 15%, denoted as Fil-s A) and the lowest (ca 5%, denoted as Fil-s B) percentage of PP measured. 

It was demonstrated that the addition of the compatibilizer inside both Fil-s batches, as such and filled with the nanosilicate, significantly affected their dynamic shear rheological properties η’ and G’, with more pronounced increments in the case of Fil-s A-based systems and, particularly, for the sample Fil-s A + 5%PE/PP-g-MA + 5%D67G. 

Concerning the transient extensional rheological behavior of the recycled materials, Fil-s B showed a slightly higher strain hardening character compared with Fil-s A due to a major content of the branched polymeric fraction. Moreover, the presence of the compatibilizer and/or the nanofiller significantly affected the tensile stress growth coefficient data, η_E_^+^, which resulted in slightly different absolute values depending on the type of Fil-s, but essentially following the same trends. In particular, the compatibilizer improved the strain hardening behavior of the recycled matrices and, when added together with the nanofiller, it caused the upward translation of the η_E_^+^ plot in the whole range of times investigated.

These observed changes in the rheological behavior of the recycled materials after the melt-compounding with the nanofiller and/or the compatibilizer were coherently correlated with their suitability for film blowing extrusion. In particular, the addition of the nanosilicate positively affected bubble stability, and the presence of the compatibilizer allowed a more uniform film deformation.

However, it is worthy to point out that the differences detected in the composition of the selected Fil-s batches inevitably influenced their processability by film blowing and the final films’ performances. First of all, a higher thermal profile had to be imposed for Fil-s A-based systems compared with Fil-s B-based ones. Furthermore, the higher compositional dis-uniformity of Fil-s A prevented the same degree of stretching set for Fil-s B systems to be achieved, resulting in thicker films.

In terms of tensile mechanical properties, Fil-s A-based films showed higher stiffness (due to a greater content of PP) and ductility (reasonably due to the higher film thickness) compared to Fil-s B-based ones. However, in the case of Fil-s B + 5%PE/PP-g-MA sample, it was possible to apply more severe stretching conditions, allowing a very thin film (30 µm) with improved stiffness (ca. 45%) and tensile strength (ca. 80%) to be produced, with respect to the corresponding thicker specimen (50 µm).

## Figures and Tables

**Table 1:** Technical features of the laboratory-scale blown film apparatus.

Single screw extruder (GIMAC, Castronno-Italy)	L/D = 24, D_screw_ = 12 mm
Blown film die TEACH-LINE (Dr. Collin GmbH, Munich-Germany)	D_in_ = 30 mm, D_ex_ = 32 mm
Film Blowing Unit BL50 TEACH-LINE (Dr. Collin GmbH, Munich-Germany)	Nip roll width 200 mm Take-off speed 1–12 m/min

**Table 2:** Operative conditions for film blowing extrusion of the different Fil-s batches.

Material	Temperature Profile from Hopper to Die (°C)	V_Collection_ (m/min)	TUR	BUR
Fil-s A	210–230–230–220	1.5	6	1.5
Fil-s B	185–190–200–185	3	13	1.5

**Table 3:** FTIR signals and vibrational assignments for Fil-s.

Wavenumbers (cm^−1^)	Assignment
3500–3200	−OH and −NH stretching
1730	C=O stretching
1600–1565	Conjugated (C=C) stretching, −NH stretching
1260	C−O stretching
1100–1000	Si−O stretching
875	C−O bending

**Table 4:** G’ slopes at low frequencies for both Fil-s A and Fil-s B systems.

Sample	G’ Slope	Sample	G’ Slope
Fil-s A	0.62	Fil-s B	0.66
Fil-s A + 5%D67G	0.61	Fil-s B + 5%D67G	0.65
Fil-s A + 5%PE/PP-g-MA	0.58	Fil-s B + 5%PE/PP-g-MA	0.63
Fil-s A + 5%PE/PP-g-MA + 5%D67G	0.49	Fil-s B + 5%PE/PP-g-MA + 5%D67G	0.54

**Table 5:** EVI values for Fil-s B-based systems.

Sample	Extensional Viscosity Index (EVI)
Fil-s B	4.8
Fil-s B + 5%D67G	4.0
Fil-s B + 5%PE/PP-g-MA	7.7
Fil-s B + 5%PE/PP-g-MA + 5%D67G	5.8

**Table 6:** Thermal data relative to the first heating scan (where T_mPE_, ΔH_mPE_, X_cPE_ and T_mPP_, ΔH_mPP_, X_cPP_ are the melting temperatures, enthalpies and crystallinity degrees of PE and PP fractions, respectively) for Fil-s A and Fil-s B-based films. The standard deviations of the thermal data were less than 5%.

FILM	T_mPE2_ (°C)	* ΔH_mPE_ (J/g)	X_cPE_ (%)	T_mPP_ (°C)	* ΔH_mPP_ (J/g)	X_cPP_ (%)
Fil-s A + 5%D67G	125	92.0	31.4	160	61.3	29.6
Fil-s A + 5%PE/PP-g-MA	125	92.7	31.6	162	64.0	30.9
Fil-s A + 5%PE/PP-g-MA + 5%D67G	125	85.0	29.0	162	58.0	28.0
Fil-s B + 5%D67G	124	82.5	28.2	161	62.0	29.9
Fil-s B + 5%PE/PP-g-MA	123	79.7	27.2	161	64.0	30.9
Fil-s B + 5%PE/PP-g-MA + 5%D67G	123	81.6	27.8	160	70.0	33.8

* The melting enthalpies (ΔH_mPE_ and ΔH_mPP_) were normalized with respect to the relative polyethylene and polypropylene contents of Fil-s A and Fil-s B.

**Table 7:** Main tensile mechanical properties (E, Young’s modulus; ε_b_ strain at break; σ_b_ stress at break) of Fil-s A and Fil-s B-based films. Regarding the films in each section, if the data in the same column are marked with different superscript letters, it means that a significant difference (*p* < 0.05) exists between the specimens.

**Section 1: Fil-s A Films (100 μm)**	**E (MPa)**	**ε_b_ (%)**	**σ_b_ (MPa)**
Fil-s A + 5%D67G	530 ± 30 ^b^	350 ± 60 ^ac^	13 ± 1 ^a^
Fil-s A + 5%PE/PP-g-MA	510 ± 20 ^b^	290 ± 30 ^a^	15 ± 1 ^b^
Fil-s A + 5%PE/PP-g-MA + 5%D67G	410 ± 20 ^a^	430 ± 90 ^bc^	16 ± 1 ^b^
**Section 2: Fil-s B Films (50 μm)**	**E (MPa)**	**ε_b_ (%)**	**σ_b_ (MPa)**
Fil-s B + 5%D67G	360 ± 30 ^c^	130 ± 10 ^a^	14 ± 1 ^a^
Fil-s B + 5%PE/PP-g-MA	270 ± 30 ^a^	140 ± 20 ^a^	15 ± 1 ^b^
Fil-s B + 5%PE/PP-g-MA + 5%D67G	330 ± 20 ^b^	120 ± 10 ^a^	13 ± 2 ^a^
**Section 3: Fil-s B Films with the** **Compatibilizer**	**E (MPa)**	**ε_b_ (%)**	**σ_b_ (MPa)**
Fil-s B + 5%PE/PP-g-MA (50 μm)	270 ± 30 ^a^	140 ± 20 ^a^	15 ± 1 ^a^
Fil-s B + 5%PE/PP-g-MA (30 μm)	390 ± 20 ^b^	130 ± 20 ^a^	27 ± 2 ^b^

## Data Availability

The data presented in this study are available on request from the corresponding author.
